# Effects of Ramadan Fasting on Intradialytic Hypoglycemia and Clinical Outcomes in Patients with Diabetes on Maintenance Hemodialysis: A Multicenter Prospective Cohort Study in Southern Thailand

**DOI:** 10.3390/medsci14050522

**Published:** 2026-08-27

**Authors:** Ashari Saman, Jayanton Patumanond, Moragot Chatatikun, Atthaphong Phongphithakchai

**Affiliations:** 1 Department of Medicine, Pattani Hospital, Pattani 94000, Thailand; azha_raul@hotmail.com; 2 Division of Clinical Epidemiology and Clinical Statistics, Faculty of Medicine, Naresuan University, Phitsanulok 65000, Thailand; jpatumanond@gmail.com; 3 Department of Medical Technology, School of Allied Health Sciences, Walailak University, Nakhon Si Thammarat 80160, Thailand; moragot.ch@wu.ac.th; 4 Nephrology Unit, Division of Internal Medicine, Faculty of Medicine, Prince of Songkla University, Songkhla 90110, Thailand

**Keywords:** Ramadan, hemodialysis, end-stage kidney disease, diabetes mellitus

## Abstract

Background: Patients with end-stage kidney disease (ESKD) and diabetes undergoing maintenance hemodialysis (HD) may be at increased risk of metabolic disturbances and intradialytic complications during Ramadan fasting. However, evidence regarding the safety of fasting in this population remains limited. This study evaluated the association between Ramadan fasting and intradialytic hypoglycemia and other clinical outcomes. Methods: This multicenter prospective cohort study included 153 patients with ESKD and diabetes undergoing maintenance HD at four centers in Thailand during the pre-Ramadan and Ramadan periods in 2024. Participants were categorized as non-fasting, partial fasting, or full fasting. All underwent HD using glucose-containing dialysate (100 mg/dL) and received standardized pre-Ramadan medication adjustments, including insulin dose reduction and oral glucose-lowering medication modification. The primary outcome was intradialytic hypoglycemia, defined as a blood glucose level <70 mg/dL. Secondary outcomes included HD-related complications, blood pressure, interdialytic weight gain (IDWG), dialysis adequacy, and laboratory parameters. Results: Among 153 participants, 73 were non-fasting, 44 were partial fasting, and 36 were full fasting. No intradialytic hypoglycemia occurred in the full- or non-fasting groups, whereas one episode (0.2%) occurred in the partial-fasting group. HD-related complications, blood pressure, IDWG, and dialysis adequacy (Kt/V) were comparable across groups. Serum potassium and parathyroid hormone levels increased during Ramadan in the full- and non-fasting groups without reported clinical events. Overall, participants remained clinically stable under the standardized protocol. Conclusions: In this cohort, Ramadan fasting was not associated with an observed increase in major intradialytic hypoglycemic events and hemodialysis-related complications, indicating that the practice is clinically feasible and well-tolerated under strictly supervised protocols. Therefore, fasting may be considered for carefully selected patients with ESKD and diabetes receiving maintenance hemodialysis, provided it is accompanied by individualized medication adjustments, meticulous monitoring, and shared decision-making.

## 1. Introduction

Fasting during Ramadan poses significant clinical challenges for hemodialysis (HD) patients with end-stage kidney disease (ESKD) and coexisting diabetes. This high-risk population is highly susceptible to complications including hypoglycemia, fluid overload, and electrolyte disturbances [1,2,3,4,5]. Despite these medical cautions, many patients are deeply motivated to observe the fast, highlighting the critical necessity for tailored, evidence-based guidance rather than absolute prohibition. While previous literature reports inconsistent findings regarding intradialytic hemodynamics and biochemical parameters, hypoglycemia remains a universally critical hazard [6,7,8,9,10,11]. In diabetic HD patients, asymptomatic hypoglycemia frequently occurs near the end of the dialysis session despite the use of glucose-supplemented dialysate, and this inherent risk is significantly exacerbated during Ramadan fasting hours [12,13,14].

While some evidence exists regarding Ramadan fasting in Southeast Asian populations, the overall data remain limited, constraining the generalizability of guidelines derived primarily from Middle Eastern cohorts. Crucially, distinct regional variables—such as a tropical climate and specific dietary customs featuring high-potassium tropical fruits (e.g., durian, papaya, mango) and carbohydrate-dense desserts—introduce unique clinical risks that are not fully addressed in the current literature. Moreover, the impact of Ramadan fasting on intradialytic glycemic excursions remains underexplored, particularly regarding the lack of systematic pre- and post-dialysis monitoring in diabetic hemodialysis patients. Bridging this evidence gap is imperative for Southern Thailand, where clinicians must navigate the delicate balance between respecting religious observances and ensuring hemodynamic and metabolic stability for an expanding demographic of Muslim ESKD patients.

Therefore, the present study aimed to prospectively assess the effect of Ramadan fasting among HD patients with ESKD and diabetes mellitus. We specifically investigated the occurrence of intradialytic hypoglycemia, alongside other adverse intradialytic events, biochemical changes, and dialysis adequacy, providing locally relevant evidence to guide clinical practice and inform recommendations for this vulnerable population.

## 2. Materials and Methods

### 2.1. Study Design and Population

This prospective cohort study was conducted in Pattani Province, southern Thailand, during the pre-Ramadan and Ramadan periods of 2024 (February–April). We recruited individuals with ESKD receiving maintenance hemodialysis (HD) who also had comorbid diabetes mellitus from four HD units: Pattani Hospital, Khok Pho Hospital, Fort Ingkhayutthaborihan Hospital, and Siroros Pattani Hospital. The study was conducted in accordance with the Declaration of Helsinki and was approved by the Institutional Review Board (IRB) of Pattani Hospital, Thailand (Approval No. PTN-011-2567; approval date: 8 March 2024).

The study population included adults aged ≥ 18 years who had been receiving maintenance HD for at least three months and had a confirmed diagnosis of diabetes mellitus. Patients were excluded if they were unable to provide informed consent or had acute medical conditions that precluded fasting. Written informed consent was obtained from all participants prior to enrollment. The cohort was categorized into three groups based on their predefined Ramadan fasting regimens: (1) full-fasting, who fasted daily, including >90% of scheduled HD days; (2) partial-fasting, who routinely fasted but deliberately excluded HD days (schedule-driven, not reactively broken due to hypoglycemia); and (3) non-fasting, who maintained their standard diets. During the study period in southern Thailand, the daily fasting duration from dawn (Suhoor) to sunset (Iftar) was approximately 13 h.

Intradialytic hypotension (IDH) was defined as a decrease in systolic blood pressure > 20 mmHg or a reduction in mean arterial pressure > 10 mmHg requiring intervention [15], while intradialytic hypertension (IDHTN) was defined as an increase in systolic blood pressure > 10 mmHg or mean arterial pressure > 15 mmHg, with absolute blood pressure exceeding 180/110 mmHg during dialysis [16]. Hyperkalemia was defined as a serum potassium level > 5 mmol/L [17], hyperphosphatemia as serum phosphate > 5 mg/dL [18], and hypoalbuminemia as serum albumin < 3.8 g/dL [19].

### 2.2. Pre-Ramadan Preparation and Clinical Management

Prior to the fasting month, the entire cohort underwent a comprehensive medication review and multidisciplinary dietary counseling to proactively mitigate fasting-related complications. In accordance with standard recommendations [4], antidiabetic regimens were preemptively adjusted through the modification of oral agents and a 15–30% prophylactic reduction in insulin doses, tailored to individualized clinical assessments. Concurrently, personalized dietary education emphasized gradual, evenly distributed fluid intake during non-fasting hours and strict potassium restriction, with specific warnings against traditional high-potassium foods such as dates and regional tropical fruits (bananas, papayas, and durians).

To maintain intradialytic glycemic and hemodynamic stability, dialysis prescriptions were dynamically tailored. Specifically, a standard glucose-containing dialysate (100 mg/dL) was universally prescribed as a continuous glycemic buffer, while ultrafiltration goals were individualized per session based on real-time bedside assessments (IDWG, hemodynamics, and volume status) rather than static historical dry weights.

### 2.3. Data Collection and Monitoring

Following the implementation of baseline risk-mitigation protocols, demographic and clinical characteristics were recorded. Throughout both the pre-Ramadan and Ramadan observation periods, patients underwent weekly evaluations comprising paired pre- and post-dialysis capillary blood glucose monitoring, continuous intradialytic blood pressure tracking, and routine laboratory assessments for biochemical parameters and dialysis adequacy.

Glucose levels were quantified via a point-of-care analyzer (Accu-Chek Instant; Roche Diabetes Care GmbH, Mannheim, Germany), with supplementary testing performed immediately if hypoglycemic symptoms occurred. To guarantee systemic glycemic equilibration, standardized post-hemodialysis finger-prick measurements were obtained 3 to 5 min following blood pump cessation. Routine laboratory samples were primarily drawn prior to the mid-week session (a 1-day interdialytic interval); however, reflecting real-world clinical practice in Thailand, patients on a twice-weekly regimen had blood drawn after a 2-day interdialytic break.

### 2.4. Study Endpoints

The primary endpoint was the occurrence of intradialytic hypoglycemia, defined as a pre- or post-hemodialysis blood glucose level <70 mg/dL, with or without clinical symptoms. Secondary endpoints included dialysis-related complications (hypotension, hypertension, muscle cramps, dizziness, and volume overload), interdialytic weight gain (IDWG), ultrafiltration (UF) volume, and changes in laboratory parameters—comprising hemoglobin, hematocrit, serum albumin, electrolytes, calcium, phosphate, intact parathyroid hormone, lipid profiles, blood urea nitrogen, and dialysis adequacy indices (urea reduction ratio [URR] and Kt/V).

### 2.5. Statistical Analysis

Baseline characteristics were summarized using descriptive statistics. Continuous variables were presented as mean ± standard deviation (SD) or median with interquartile range (IQR), depending on data distribution, while categorical variables were expressed as counts and percentages. Between-group comparisons were performed using one-way analysis of variance (ANOVA) or Kruskal–Wallis tests for continuous variables, and chi-square or Fisher’s exact tests for categorical variables, as appropriate.

Longitudinal outcomes, including blood pressure, interdialytic weight gain, blood glucose, and ultrafiltration volume across Ramadan weeks, were analyzed using generalized estimating equations (GEEs) to account for within-patient correlation over repeated measures. For categorical or count outcomes (e.g., intradialytic hypotension, muscle cramps), generalized linear models with Poisson regression and robust standard errors were applied, with clustering at the patient level to adjust for repeated observations. To account for baseline heterogeneity and mitigate potential confounding, multivariable Generalized Linear Models were constructed for longitudinal intradialytic outcomes. All analyses were performed using Stata version 16.0 (StataCorp, College Station, TX, USA).

## 3. Results

### 3.1. Baseline Characteristics

A total of 153 patients with ESKD and diabetes mellitus were enrolled: 36 (24%) in the full fasting group, 44 (29%) in the partial fasting group, and 73 (47%) in the non-fasting group. Baseline characteristics are presented in Table 1. The mean age was comparable across groups (56.9, 58.1, and 60.2 years; *p* = 0.34). Male participants constituted 58.3% of the full fasting group, 40.9% of the partial fasting group, and 39.7% of the non-fasting group (*p* = 0.16). Dialysis duration was significantly shorter in the partial fasting group (median 13 months) compared with the full fasting (20 months) and non-fasting groups (24 months, *p* = 0.028). Hypertension was the most common comorbidity (>80% across all groups), while diabetes mellitus was the leading cause of ESKD (>90%). Mean HbA1c was significantly higher in the full fasting group (7.4 ± 2.0) compared with the partial fasting (6.3 ± 1.5) and non-fasting (7.0 ± 1.7) groups (*p* = 0.010).

### 3.2. Hypoglycemia

Prior to Ramadan, post-hemodialysis hypoglycemia was extremely rare, with only a single event observed in the non-fasting cohort (0.3%). During Ramadan, hypoglycemic events remained negligible, occurring exclusively as a single episode in the partial-fasting group (0.2%), with zero episodes in both the full-fasting and non-fasting groups. In unadjusted analysis, hypoglycemia incidence did not differ significantly between the full-fasting and non-fasting groups before or during Ramadan (*p* = 0.408 and *p* > 0.999, respectively; Table 2). Multivariable-adjusted analysis confirmed no significant disparity in hypoglycemia risk prior to Ramadan (adjusted *p* = 0.633), whereas formal model estimation during Ramadan was precluded by the absence of events in the reference cohorts (Table 3).

Patient distribution across hemodialysis shifts—morning (Shift 1), mid-day (Shift 2), and afternoon/evening (Shift 3)—was homogeneous across cohorts throughout the study (all *p* > 0.05), with 42–48% of patients receiving morning dialysis weekly. Shift-specific hypoglycemia occurred exclusively during Shift 1 pre-Ramadan (non-fasting group) and Shift 2 during Ramadan (partial-fasting group), with zero events during Shift 3.

**Figure 1:**
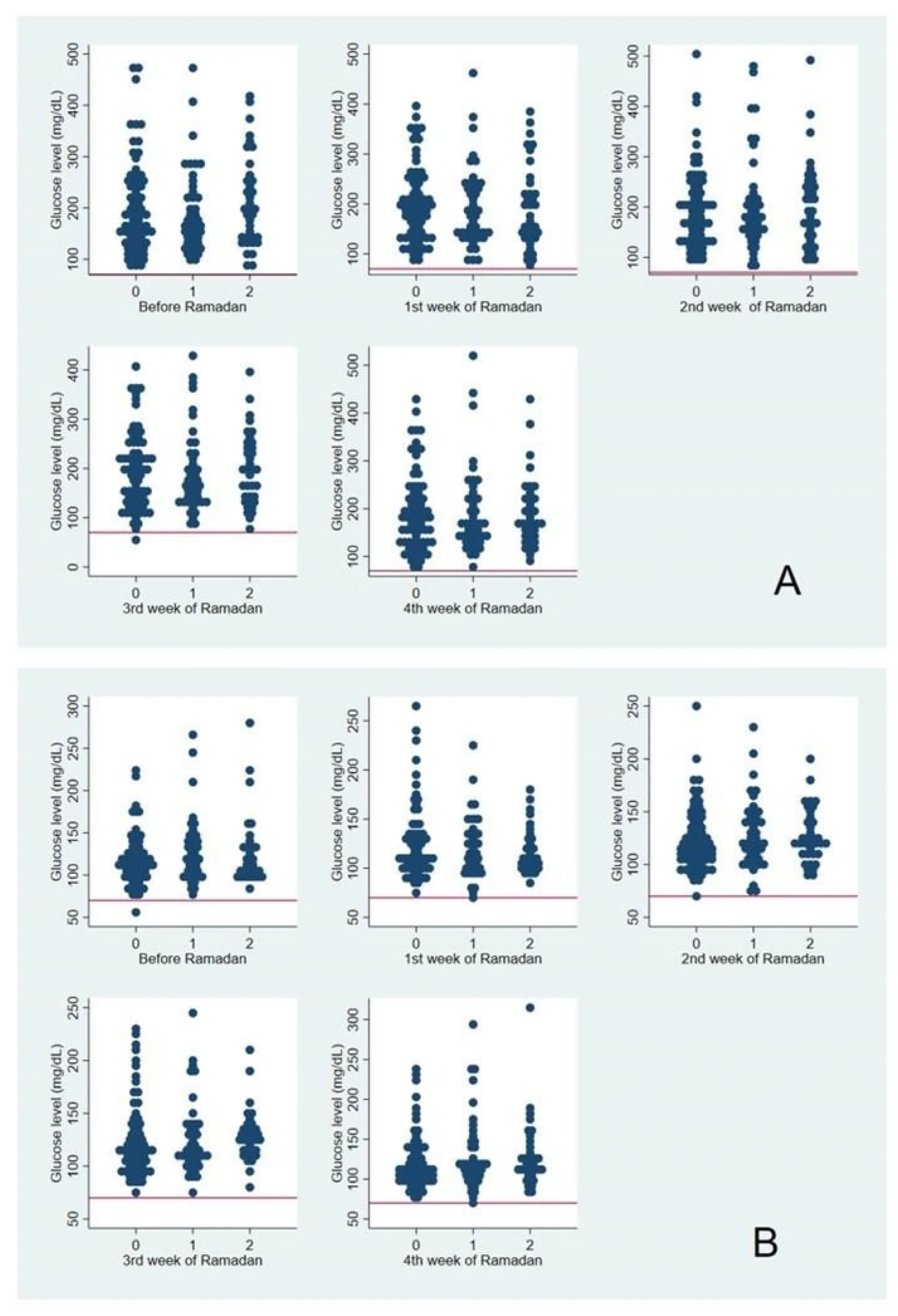
**Pre-hemodialysis** (**A**) **and post-hemodialysis** (**B**) **blood glucose levels before and across the four weeks of Ramadan.** Group designations on the x-axis denote the fasting status: 0 = non-fasting group (n = 73), 1 = partial-fasting group (n = 44), and 2 = full-fasting group (n = 36). Dots represent individual glucose measurements. The patient count for each respective cohort remained constant across all measurement time points. The solid horizontal reference line indicates the standard hypoglycemic threshold of 70 mg/dL.

Overall glycemic control remained stable: pre-dialysis glucose showed no significant weekly shifts across cohorts (*p* = 0.154–0.870), and post-dialysis glucose remained unchanged in the full-fasting cohort (*p* = 0.171–0.870). Minor Week 1 post-dialysis glucose fluctuations in partial-fasters (β = −8.07 mg/dL; *p* = 0.031) and non-fasters (β = 8.10 mg/dL; *p* = 0.026) completely resolved from Week 2 onward (all *p* > 0.05) (Figure 1).

### 3.3. Dialytic Complications and Blood Pressure

During Ramadan, the incidence of intradialytic hypotension (IDH) occurred in 4.0%, 4.5%, and 3.5% of patients in the full-fasting, partial-fasting, and non-fasting groups, respectively, with no statistically significant difference between the full-fasting and non-fasting cohorts (adjusted *p* = 0.892). Similarly, intradialytic hypertension (IDHTN) was observed in 0.9%, 2.0%, and 0.7% of patients, respectively (full-fasting vs. non-fasting: adjusted *p* = 0.666). The incidence of volume overload was likewise comparable across groups (0.3%, 1.8%, and 0.4%, respectively; adjusted *p* = 0.682). Other reported intradialytic symptoms, including dizziness (adjusted *p* = 0.846) and muscle cramps (adjusted *p* = 0.623), did not differ significantly among the study groups (Table 2 and Table 3).

**Figure 2:**
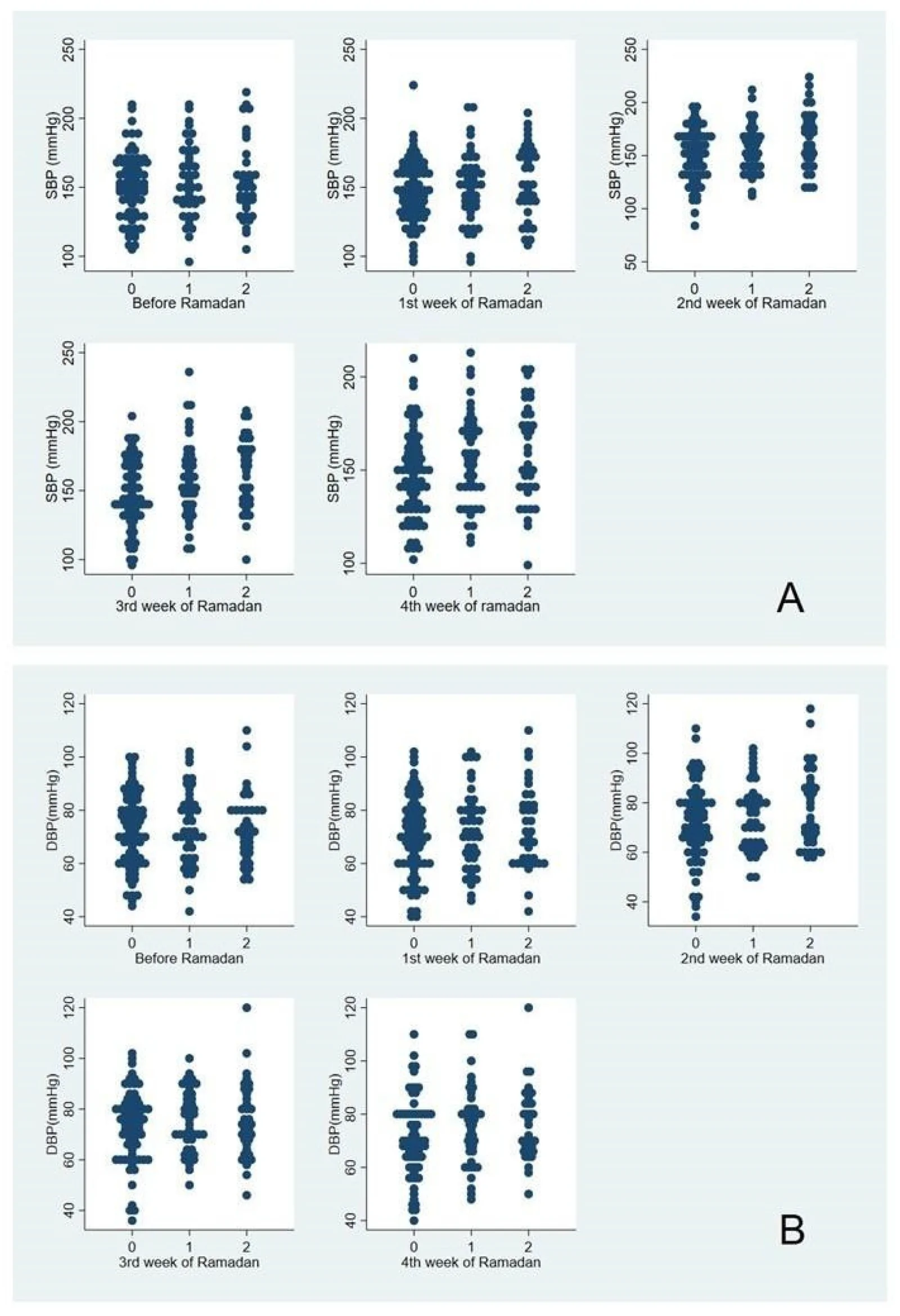
**Pre-dialysis systolic** (**A**) **and diastolic** (**B**) **blood pressure trends before and during the Ramadan period.** The x-axis categorizes the patient cohorts based on fasting behavior: 0 = non-fasting group (n = 73), 1 = partial-fasting group (n = 44), and 2 = full-fasting group (n = 36). Dots represent individual blood pressure measurements. The number of patients included in each group was consistent at every designated measurement time point.

Trends in pre-dialysis systolic and diastolic blood pressure are illustrated in Figure 2. Longitudinal blood pressure profiles remained generally stable relative to baseline across all cohorts. In the full-fasting group, mean systolic blood pressure (SBP) showed a transient, modest elevation at Week 2 (β = 10.92 mmHg; 95% CI, 0.90 to 20.94; *p* = 0.033), but returned to baseline in Weeks 3 and 4 (*p* = 0.220 and *p* = 0.361, respectively). No other significant SBP fluctuations occurred in the partial-fasting (*p* = 0.154–0.642) or non-fasting groups (*p* = 0.062–0.520). Similarly, diastolic blood pressure trajectories showed no significant deviations across all four weeks of Ramadan across cohorts (all *p* > 0.05).

### 3.4. Laboratory Findings

Changes in laboratory parameters are summarized in Table 4. Serum potassium levels increased significantly in both the full-fasting (4.6 ± 0.7 to 4.8 ± 0.7 mmol/L, *p* = 0.012) and non-fasting groups (4.5 ± 0.7 to 4.7 ± 0.9 mmol/L, *p* = 0.004). Similarly, parathyroid hormone (PTH) levels rose significantly in the full-fasting (median 369 [IQR 189–525] to 414 [238–660], *p* = 0.013) and non-fasting groups (254 [118–544] to 280 [157–481], *p* = 0.007). Furthermore, the non-fasting group exhibited significant increases in serum albumin (3.8 ± 0.5 to 3.9 ± 0.4 g/dL, *p* = 0.001) and blood urea nitrogen (BUN) (56 ± 15.6 to 60 ± 16.3mg/dL, *p* = 0.028). Other parameters, including hemoglobin, calcium, phosphate, cholesterol, URR, and Kt/V, remained stable across all groups.

### 3.5. Dialysis Parameters

**Figure 3:**
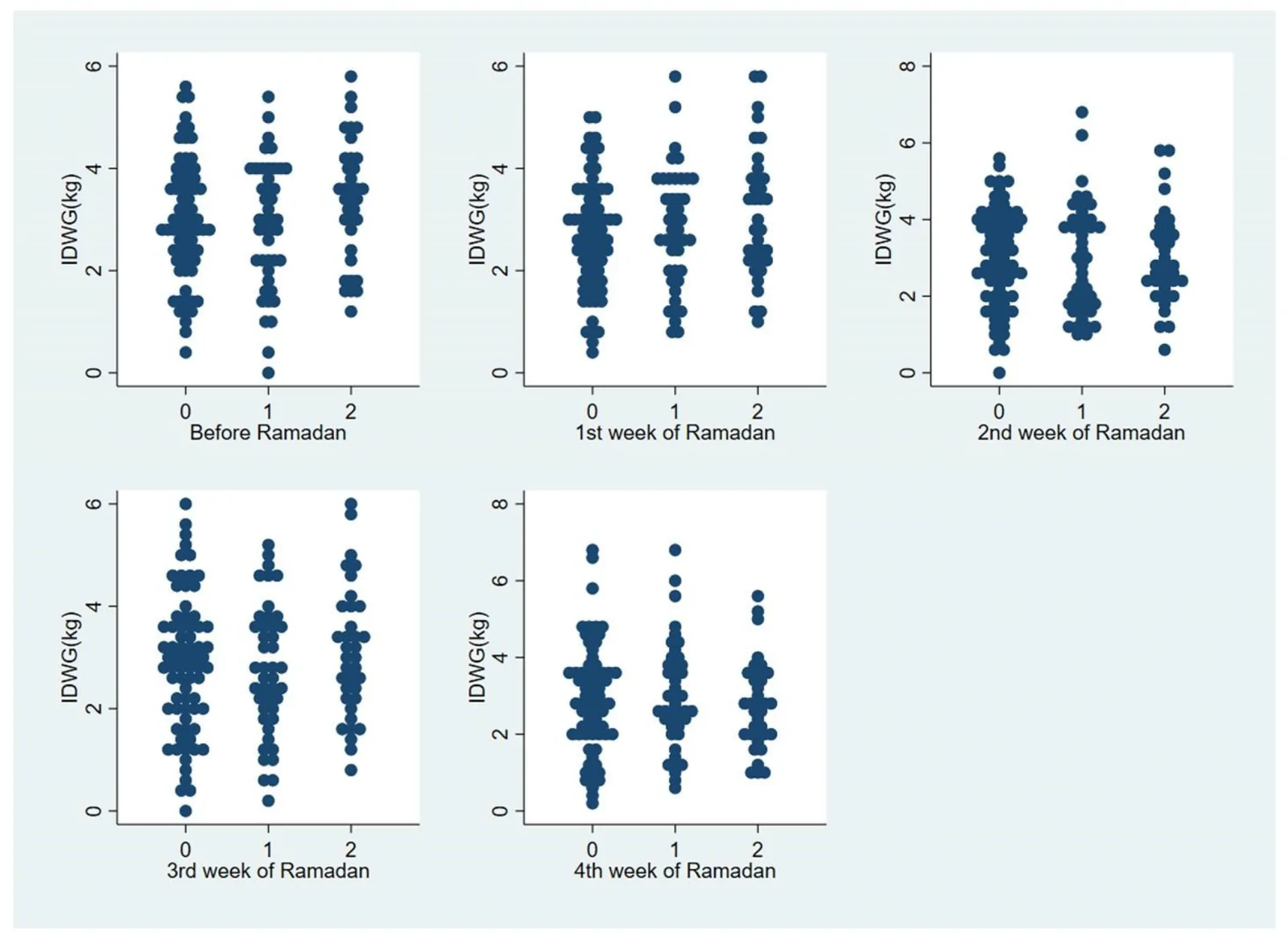
**Interdialytic weight gain (IDWG) distribution before and during Ramadan.** Participants are stratified on the x-axis according to their fasting adherence: 0 = non-fasting group (n = 73), 1 = partial-fasting group (n = 44), and 2 = full-fasting group (n = 36). Dots represent individual IDWG measurements. The sample size for each specific group remained unchanged across all weekly measurement time points.

Interdialytic weight gain (IDWG) and ultrafiltration (UF) volumes remained generally stable throughout Ramadan relative to pre-Ramadan baselines across all cohorts. In the full-fasting group, IDWG decreased significantly at Week 4 (β = −0.63 kg; *p* = 0.003), with stable UF volumes throughout (*p* = 0.091). The partial-fasting group maintained stable IDWG across all weeks (*p*= 0.388), alongside a minor UF volume decline at Week 3 (β = −0.29 L; *p* = 0.037). The non-fasting group experienced minor transient dips in Week 1 IDWG (β = −0.32 kg; *p* = 0.042) and UF volumes in Weeks 1 and 4 (β = −0.25 L and −0.22 L; *p* = 0.015 and 0.045, respectively) (Figure 3).

## 4. Discussion

This prospective cohort study demonstrates that Ramadan fasting is clinically feasible for Thai patients with diabetes undergoing maintenance HD, provided it is managed through supervised medication adjustment and the use of glucose-containing dialysate. Regarding the primary endpoint, both the full-fasting and non-fasting groups remained free of hypoglycemic events during the holy month. While hypoglycemia was documented exclusively within the partial-fasting cohort, these episodes were rare and devoid of immediate adverse clinical sequelae, indicating that the acute risk appears manageable under strict clinical supervision.

We observed stability in key secondary outcomes, such as intradialytic complications, IDWG, and Kt/V, across all study arms. Although laboratory analysis revealed subtle increases in BUN and albumin among non-fasting patients, as well as in potassium and PTH levels across both full-fasting and non-fasting groups, these variations were numerically small and lacked clinical significance. Blood pressure trends were comparable across all groups before and during Ramadan. Overall, fasting was not associated with an observed increase in adverse clinical outcomes, suggesting that Ramadan fasting appears feasible for high-risk populations under the structured management protocol used in this study.

Our findings align with, but significantly refine, the established literature regarding the risks of fasting in maintenance HD patients. As summarized in Supplementary Table S1, prior observational studies from Southeast Asia and the Middle East present highly variable incidences of intradialytic complications and hypoglycemia. A critical synthesis of these disparities highlights several distinct methodological and demographic divergences between our study and historical cohorts. First, regarding patient selection, our cohort was exclusively diabetic (100%), whereas previous major studies comprised mixed populations with substantially lower diabetes prevalence (ranging from 11.6% to 54.5%) [7,8,9,10,20,21]. Despite this inherently higher-risk demographic, intradialytic hypoglycemia was not observed in either the full-fasting or non-fasting cohorts, with an exceptionally low incidence (0.2%) confined exclusively to the partial-fasting group. This contrasts sharply with prior reports, such as a Malaysian cohort where hypoglycemia surged to 53% among diabetic fasters [7] and other cohorts reporting subjective symptomatic events in 10% to 15.7% of fasting patients [8,20].

We hypothesize that this notable discrepancy in hypoglycemia incidence is multifactorial, potentially reflecting differences in clinical management and glucose-monitoring strategies. First, proactive pre-Ramadan regimen optimization (oral antidiabetic adjustments and a preemptive 15–30% insulin reduction) may have served as a key mitigating factor against hypoglycemic events, although our observational design strictly precludes establishing definitive causality. Second, the universal adoption of 100 mg/dL glucose dialysate theoretically provided a continuous intradialytic glycemic buffer, which might have protected against sharp glucose nadirs. Third, baseline glycemic variations across cohorts (HbA1c: 7.4% in full-fasters, 7.0% in non-fasters, and 6.3% in partial-fasters; *p* = 0.010) plausibly offered a broader physiological cushion before reaching hypoglycemic thresholds. Fourth, treatment timing emerges as a potential protective factor, given that 42–48% of patients underwent morning dialysis shortly after the pre-dawn meal (Suhoor). This brief postprandial interval, coupled with the gradual digestion of complex carbohydrates, plausibly provided a physiological buffer against acute intradialytic glucose nadirs. Importantly, the distribution of dialysis shifts was homogeneous across all cohorts, thereby minimizing shift timing as a potential confounder. Future multicenter studies incorporating shift-stratified risk analyses are warranted to further elucidate these specific dietary and pharmacokinetic dynamics. Finally, our diagnostic approach utilized objective, biochemical capillary glucose monitoring at precisely defined intervals, offering a distinct methodological advantage over prior literature that relied predominantly on retrospective, defining hypoglycemia via subjective symptom recall.

However, a notable limitation of our protocol is the lack of continuous glucose monitoring (CGM). Intermittent glucose testing restricted to pre- and post-dialysis timepoints inherently limits the detection of transient, asymptomatic intradialytic hypoglycemia that may occur mid-session. Although the continuous glucose concentration in the 100 mg/dL dialysate fluid likely served as a physiological buffer against sharp glucose nadirs—and no post-dialysis hypoglycemic events were recorded—isolated intradialytic episodes could remain undetected without continuous sensing. Consequently, while our findings demonstrate the clinical feasibility and overall tolerability of managed fasting, absolute exclusion of asymptomatic intradialytic hypoglycemia warrants confirmation through CGM-integrated prospective trials.

Consistent with historical studies in Egypt (3.4% hypotension/cramps) [21] and Malaysia (45% to 35% hypotension reduction) [7], intradialytic complications remained low during Ramadan. IDH incidence in full-fasters (4.0%) was comparable to non-fasters (3.5%; adjusted *p* = 0.892; Table 3). After adjusting for baseline HbA1c, dialysis duration, and alpha-blocker use, no significant intergroup variations occurred in intradialytic hypertension, volume overload, dizziness, or cramping (all adjusted *p* > 0.05). Collectively, our findings indicate that no significant increase in the risk of acute intradialytic adverse events was detected in this cohort while adhering to a managed fasting protocol.

Regional and environmental contexts, including fasting duration and dietary habits, introduce significant biochemical heterogeneity. Our tropical cohort fasted for approximately 13 h, whereas Middle Eastern cohorts often faced 15 to 16 h fasts [8,9]. This duration, coupled with culturally distinct Southeast Asian Iftar habits (e.g., carbohydrate-dense desserts and potassium-rich tropical fruits like durian and papaya), yields differing metabolic responses compared to diets in the Middle East or Western nations. Consequently, the modest albumin rise in our non-fasting group contrasts with variable global trends [7,20,22,23], while our stable serum phosphate levels diverge from fluctuations observed in Middle Eastern [8,10] and Malaysian [7] cohorts. Furthermore, the significant hyperkalemia observed in our fasting cohorts aligns with reports from Pakistan [24], Malaysia [20], and Palestine [9], yet contrasts with findings of potassium stability elsewhere [10,22]. Mechanistically, post-Iftar intake of potassium-dense foods may coincide with the 15–30% insulin reduction prophylactically instituted for hypoglycemia prevention. In theory, this reduced insulin availability may attenuate intracellular potassium buffering. Future research incorporating digital food diaries, CGM, and precise medication logs is required to further delineate the relative contributions of dietary intake versus insulin adjustments on potassium balance.

In our study, fluid status and hemodynamic parameters remained stable, with no significant differences in IDWG or blood pressure. These findings align with reports from Malaysia [7], Pakistan [22], and Saudi Arabia [10]. Conversely, regional variability exists; other cohorts reported fluctuations ranging from reduced dry weight in Egypt [21] to increased IDWG in Palestine [9].

These findings offer pivotal clinical implications for managing hemodialysis patients during Ramadan. First, fasting may be feasible for carefully selected individuals within a shared decision-making framework. Rather than imposing rigid prohibitions—which patients often disregard due to spiritual priorities—respecting patient autonomy through structured medical oversight mitigates the risks of unmonitored fasting [25,26]. Second, successful fasting requires individualized management, encompassing strict dietary and fluid counseling (specifically limiting potassium and phosphate intake) [5,27,28] alongside proactive, protocol-driven regimens (preemptive medication modifications and glucose-containing dialysate) to maintain glycemic stability. Third, pre-Ramadan risk stratification aligned with international guidelines (e.g., IDF-DAR) remains essential to identify high-risk individuals requiring intensified surveillance or medical exemption [2,29,30].

The primary strength of this study is its prospective design, which enabled systematic monitoring of glycemic profiles during the critical peri-dialytic period—a window of peak physiological stress. By focusing on the underrepresented diabetic ESKD population within a tropical, Muslim-majority setting, our findings provide ecologically valid evidence to guide clinical management in regions facing similar climatic and dietary challenges.

Several limitations exist. First, while our study utilized a multicenter design, the cohort was exclusively drawn from a single province in southern Thailand. This geographic restriction limits the generalizability of our findings to populations with differing sociocultural and environmental contexts. Second, the observational design introduced substantial self-selection bias, reflected in baseline disparities in dialysis vintage and HbA1c. Patients choosing full fasting likely constituted a healthier, more motivated subpopulation. This unmeasured confounding—a ‘healthy faster effect’—could independently drive the favorable outcomes and mask underlying vulnerabilities. Additionally, the elevated baseline HbA1c in this cohort likely acted as a physiological buffer against hypoglycemia. Third, as noted above, reliance on pre- and post-dialysis point-of-care testing rather than CGM constrained our capacity to detect dynamic intradialytic glycemic excursions. Incorporating CGM, active post-dialysis telephone surveillance, or real-time symptom-reporting systems in future trials is essential to fully characterize 24 h interdialytic glycemic dynamics. Fourth, routine liver function tests were not systematically performed to biochemically assess hepatic health; however, no patients with known chronic liver disease or cirrhosis were included in this cohort, minimizing the likelihood of impaired hepatic gluconeogenesis as an unmeasured confounder. Fifth, although the low incidence of intradialytic hypoglycemia indicates good clinical tolerability, this low event rate inherently restricts the statistical power to formally demonstrate equivalence or exclude subtle risks. Furthermore, because these favorable outcomes were achieved under a highly structured management protocol, these findings should be interpreted with caution and may not directly apply to unmonitored fasting environments. Finally, the short study duration precludes assessing long-term sequelae.

## 5. Conclusions

In this prospective cohort of individuals with ESKD and concurrent diabetes, Ramadan fasting was not associated with an observed increase in major intradialytic hypoglycemic events. Furthermore, intradialytic complications, hemodynamic parameters, and laboratory profiles remained comparable across all study groups. While the low event rate precludes definitive claims of absolute safety, fasting appears clinically feasible and well tolerated under strictly supervised conditions. Consequently, fasting may be considered for carefully selected patients when supported by a structured management strategy, including strict counseling, preemptive dose reduction for antidiabetic agents and insulin, and glucose-containing dialysate. Given the modest sample size, larger multicenter studies are warranted to validate these observations and evaluate long-term clinical implications.

## Figures and Tables

**Table 1:** Baseline characteristics.

Variables	Full Fasting	Partial Fasting	Non-Fasting	*p* Value
	*n* = 36	*n* = 44	*n* = 73	
Age (years), Mean (±SD)	56.9 (±10.2)	58.1 (±8.8)	60.2 (±13.1)	0.342
Sex				0.158
Male	21 (58.3)	18 (40.9)	29 (39.7)	
Female	15 (41.7)	26 (59.1)	44 (60.3)	
BMI (kg/m^2^), Mean (±SD)	24.6 (±4.3)	23.4 (±3.7)	23.9 (±4.4)	0.457
Duration of dialysis (month) median (IQR)	20.0 (8–63)	13.0 (6.5–22)	24.0 (10–54)	0.028
Comorbidities				
Hypertension	29 (80.6)	38 (86.4)	65 (89.0)	0.480
Cerebrovascular disease	4 (11.1)	3 (6.8)	9 (12.3)	0.634
Cardiovascular disease	2 (5.6)	6 (13.6)	10 (13.7)	0.417
Peripheral arterial disease	2 (5.6)	2 (4.5)	2 (2.7)	0.752
Cause of ESKD				0.390
DM	33 (91.7)	43 (97.7)	71 (97.3)	
Hypertension	2 (5.6)	1 (2.3)	2 (2.7)	
Other	1 (2.8)	0 (0)	0 (0)	
HbA1c (%), Mean (±SD)	7.4 (±2.0)	6.3 (±1.5)	7.0 (±1.7)	0.010
Anti-hyperglycemic medication				
Insulin	14 (38.9)	10 (22.7)	26 (35.6)	0.245
Sulfonylureas	9 (25.0)	11 (25.0)	10 (13.7)	0.208
Pioglitazone	5 (13.9)	8 (18.2)	7 (9.6)	0.386
DPP-4 inhibitor	9 (25.0)	10 (22.7)	16 (21.9)	0.968
Anti-hypertensive medication				
ACEI	7 (19.4)	4 (9.1)	6 (8.2)	0.223
ARB	9 (25.0)	9 (20.5)	7 (9.6)	0.072
CCB	26 (72.2)	33 (75.0)	54 (74.0)	0.971
Beta blocker	10 (27.8)	9 (20.5)	14 (19.2)	0.623
Alpha blocker	12 (33.3)	11 (25.0)	9 (12.3)	0.027
Vasodilator	11 (30.6)	15 (34.1)	29 (39.7)	0.608
Centrally α_2_-agonist	3 (8.3)	8 (18.2)	9 (12.3)	0.472

Data are presented as number (percentage), or mean ± standard deviation. Abbreviations, ACEI; angiotensin-converting enzyme inhibitors, ARB; angiotensin receptor blockers, BMI; body mass index, CCB; calcium channel blocker, DM; diabetes mellitus, DPP-4; Dipeptidyl peptidase-4, ESKD; end-stage kidney disease.

**Table 2:** Incidence of adverse events and intradialytic complications before and during Ramadan across hemodialysis cohorts.

Variable	Full Fasting	Partial Fasting	Non-Fasting	*p* Value (Full vs. Non-Fasting)
	n (%)	n (%)	n (%)	
Hypoglycemia				
Before Ramadan	0 (0)	0 (0)	1 (0.3)	0.408
During Ramadan	0 (0)	1 (0.2)	0 (0)	1.000
Intradialytic hypotension				
Before Ramadan	5 (2.9)	8 (3.6)	11 (3.1)	0.903
During Ramadan	14 (4.0)	20 (4.5)	25 (3.5)	0.676
Intradialytic hypertension				
Before Ramadan	1 (0.6)	9 (4.1)	1 (0.3)	0.788
During Ramadan	3 (0.9)	9 (2.0)	5 (0.7)	0.831
Volume Overload				
Before Ramadan	2 (1.1)	4 (1.8)	1 (0.2)	0.364
During Ramadan	1 (0.3)	8 (1.8)	3 (0.4)	0.112
Dizziness				
Before Ramadan	0 (0)	3 (1.4)	1 (0.3)	0.678
During Ramadan	3 (0.9)	7 (1.6)	5 (0.7)	0.801
Cramping				
Before Ramadan	0 (0)	1 (0.5)	1 (0.3)	0.558
During Ramadan	4 (1.1)	4 (0.9)	6 (0.8)	0.620

Data are presented as total number of events (incidence rate as a percentage of total hemodialysis sessions.

**Table 3:** Multivariable-adjusted incidence rates of adverse events and intradialytic complications before and during Ramadan across hemodialysis cohorts.

Variable	Full Fasting	Partial Fasting	Non-Fasting	Adjusted Risk Difference (95% CI)	Adjusted *p*-Value
	n (%)	n (%)	n (%)		(Full vs. Non-Fasting)
Hypoglycemia					
Before Ramadan	0 (0)	0 (0)	1 (0.3)	+0.97% (−3.00% to +4.94%)	0.633
During Ramadan	0 (0)	1 (0.2)	0 (0)	— †	— †
Intradialytic hypotension					
Before Ramadan	5 (2.9)	8 (3.6)	11 (3.1)	+3.88% (−13.68%to +21.43%)	0.665
During Ramadan	14 (4.0)	20 (4.5)	25 (3.5)	−2.36% (−36.42% to +31.69%)	0.892
Intradialytic hypertension					
Before Ramadan	1 (0.6)	9 (4.1)	1 (0.3)	−0.26% (−5.82% to +5.30%)	0.927
During Ramadan	3 (0.9)	9 (2.0)	5 (0.7)	+3.15% (−11.17% to +17.48%)	0.666
Volume Overload					
Before Ramadan	2 (1.1)	4 (1.8)	1 (0.2)	−3.10% (−11.91% to +5.72%)	0.491
During Ramadan	1 (0.3)	8 (1.8)	3 (0.4)	+1.66% (−6.28% to +9.60%)	0.682
Dizziness					
Before Ramadan	0 (0)	3 (1.4)	1 (0.3)	+1.37% (−2.64% to +5.38%)	0.504
During Ramadan	3 (0.9)	7 (1.6)	5 (0.7)	−1.05% (−11.73% to +9.62%)	0.846
Cramping					
Before Ramadan	0 (0)	1 (0.5)	1 (0.3)	+1.30% (−2.73% to +5.32%)	0.528
During Ramadan	4 (1.1)	4 (0.9)	6 (0.8)	−3.63% (−18.13% to +10.86%)	0.623

Data are presented as total number of events (incidence rate as a percentage of total hemodialysis sessions). Models were adjusted for baseline HbA1c, dialysis duration, and the use of alpha-blockers. † Model failed to converge due to zero event counts.

**Table 4:** Comparison of laboratory parameters measured before and during Ramadan.

Variable	Pre-Ramadan	During Ramadan	*p* Value
Hemoglobin (g/dL)			
Full fasting	9.8 (±1.5)	10.1 (±1.2)	0.166
Partial fasting	9.3 (±1.3)	9.6 (±1.4)	0.038
Non-fasting	9.7 (±1.4)	9.8 (±1.3)	0.274
Hematocrit (%)			
Full fasting	30.1 (±4.9)	30.5 (±3.8)	0.503
Partial fasting	28.3 (±4.1)	28.9 (±4.5)	0.165
Non-fasting	29.6 (±4.3)	29.9 (±4.3)	0.306
Platelet (×10^3^ μL)			
Full fasting	217 (±71.2)	223 (±67.9)	0.341
Partial fasting	232 (±65.2)	225 (±76.6)	0.320
Non-fasting	222 (±60.5)	223 (±79.3)	0.943
Albumin (g/dL)			
Full fasting	3.9 (±0.3)	4.00 (±0.2)	0.376
Partial fasting	3.8 (±0.3)	3.9 (±0.3)	0.055
Non-fasting	3.8 (±0.5)	3.9 (±0.4)	0.001
Blood urea nitrogen (mg/dL)			
Full fasting	57 (±17.5)	59 (±16.5)	0.581
Partial fasting	47 (±15.2)	50 (±18.3)	0.205
Non-fasting	56 (±15.5)	60 (±16.2)	0.028
Urea reduction ratio (%)			
Full fasting	75.6 (±7.4)	74.4 (±8.9)	0.229
Partial fasting	77.0 (±7.8)	75.6 (±8.9)	0.261
Non-fasting	76.9 (±7.5)	76.78 (±7.6)	0.857
Kt/V			
Full fasting	1.7 (±0.3)	1.7 (±0.4)	0.149
Partial fasting	1.8 (±0.4)	1.80 (±0.3)	0.210
Non-fasting	1.8 (±0.3)	1.8 (±0.3)	0.740
Sodium (mmol/L)			
Full fasting	136 (±3.8)	136 (±2.8)	0.966
Partial fasting	137 (±3.0)	136 (±2.8)	0.007
Non-fasting	136 (±3.3)	137 (±3.2)	0.320
Potassium (mmol/L)			
Full fasting	4.6 (±0.7)	4.8 (±0.7)	0.012
Partial fasting	4.3 (±0.7)	4.4 (±0.9)	0.115
Non-fasting	4.5 (±0.6)	4.7 (±0.9)	0.004
Bicarbonate (mmol/L)			
Full fasting	21 (±3.3)	20 (±3.0)	0.367
Partial fasting	21 (±3.2)	20 (±3.0)	0.101
Non-fasting	21 (±3.2)	20 (±3.1)	0.020
Calcium (mg/dL)			
Full fasting	8.6 (±0.9)	8.7 (±1.0)	0.523
Partial fasting	8.6 (±1.2)	8.6 (±1.0)	0.679
Non-fasting	8.7 (±0.9)	8.7 (±0.1)	0.705
Phosphate (mg/dL)			
Full fasting	5.4 (±1.9)	5.7 (±1.9)	0.286
Partial fasting	5.2 (±2.0)	5.0 (±2.1)	0.463
Non-fasting	5.3 (±1.8)	5.3 (±1.6)	0.995
Cholesterol (mg/dL)			
Full fasting	166 (±48.7)	174 (±51.5)	0.348
Partial fasting	171 (±52.11)	181 (±63.4)	0.289
Non-fasting	157 (±47.5)	160 (±58.1)	0.648
Parathyroid hormone (pg/mL) median (IQR)			
Full fasting	369 (189–525)	414 (238–660)	0.013
Partial fasting	299 (183–448)	308 (171–410)	0.907
Non-fasting	254 (118–544)	280 (157–481)	0.007

Data are presented as mean ± standard deviation.

## Data Availability

The original contributions presented in this study are included in the article. Further inquiries can be directed to the corresponding author.

## Supplementary Materials

The following supporting information can be downloaded at: https://www.mdpi.com/article/10.3390/medsci14050522/s1, Table S1: Summary of Major Published Studies Evaluating Ramadan Fasting in Maintenance Hemodialysis Patients.

## Abbreviations

The following abbreviations are used in this manuscript:

ACEI	Angiotensin-converting enzyme inhibitor
ARB	Angiotensin receptor blocker
BMI	Body mass index
BUN	Blood urea nitrogen
CCB	Calcium channel blocker
CGM	Continuous glucose monitoring
DPP-4	Dipeptidyl peptidase-4
ESKD	End-stage kidney disease
HD	Hemodialysis
IDH	Intradialytic hypotension
IDWG	Interdialytic weight gain
PTH	Parathyroid hormone
UF	Ultrafiltration
URR	Urea reduction ratio
