# Supplementary material for: Effects of Ramadan Fasting on Intradialytic Hypoglycemia and Clinical Outcomes in Patients with Diabetes on Maintenance Hemodialysis: A Multicenter Prospective Cohort Study in Southern Thailand
Source: Med Sci (Basel). 2026 Aug 27;14(5):522. doi: 10.3390/medsci14050522 (PMC13609217; doi:10.3390/medsci14050522)
Supplement: Supplementary file 1 [file medsci-14-00522-s001.zip › medsci-4470938-supplementary.pdf]

Supplementary Table S1. Summary of Major Published Studies Evaluating Ramadan Fasting in Maintenance Hemodialysis Patients

Study /country	Study Design	Sample size(N)	DM (%)	Fasting pattern /Daily fasting duration	Glucose Monitoring strategy	Medication & dialysate adjustment	Hypoglycemia event	Key Clinical & Adverse Outcomes
Present Study (2026)/ Thailand	Prospective multicenter cohort study<br>(4 centers)	N = 153<br>-Full fasting:<br>(n = 36)<br>-Partial fasting:<br>n = 44<br>-Non-fasting:<br>n = 73	100%	1.Full fasting (>90% including HD days)<br>2.Partial fasting (only non -HD days)<br>3.Non-fasting<br>/13 hour	- Capillary blood glucose monitoring: pre- and post-HD, PRN for symptoms<br>- Self-reported symptomatic recall	Standardized management protocol:<br>-Preemptive 15%-30% insulin dose reduction<br>-Oral hypoglycemic agent titration<br>-Dialysate glucose concentration of 100 mg/dL	-No hypoglycemia event in full-fasting<br>- 1 event (0.2%) in partial-fasting	- No severe adverse events<br>-Stable BP, IDWG, and Kt/V<br>- Mild hyperkalemia and increase PTH level in full and non- fasting group
Wan Md Adnan et al. (2014)/Malaysia	Prospective observational cohort study<br>(3 centers)	N = 35 (voluntary fasters)	48.6%	Voluntary fasting<br>/14 hour	-Self-reported symptomatic recall<br>- pre/end Ramadan fructosamine/<br>HbA1c	Not reported<br>(no protocol specified)	-Hypoglycemia in DM patients pre-Ramadan 29.4% vs 52.9% during Ramadan<br>- 0% in non-diabetic	-Significant reduction in body weight and ultrafiltration<br>-IDH decreased from 45% to 35%<br>-Significant rise in albumin and fall in Po4<br>-Stable IDWG, BP, and K
Imtiaz et al. (2016)/Pakistan	Prospective cohort study (single center)	N = 282<br>-Fasters: 12.1%<br>-Non-fasters:<br>87.9%	Not reported	Fasting on non-HD days only (>10 days)<br>/duration not report	Not reported<br>(no protocol specified)	Not reported<br>(no protocol specified)	Not reported	- Significant rise in albumin in fasters<br>-Stable body weight, BP, K, and PO4
Alshamsi et al. (2016)/Saudi Arabia	Prospective multicenter cross-sectional<br>(5 centers)	N = 635<br>(fasters: 64.1%)	54.5%	Fasting vs<br>Non-fasting<br>/12-14 hour fasting	Not reported<br>(no protocol specified)	Not reported<br>(no protocol specified)	Not reported	-Higher PO4 in fasters<br>- Stable BP, DW, IDWG, K, albumin, & IDH rates

Study /country	Study Design	Sample size	DM (%)	Fasting pattern /Daily fasting duration	Glucose monitoring strategy	Medication & dialysate adjustment	Hypo- glycemia event	Key Clinical & Adverse Outcomes
Khazneh et al. (2019)/ Palestine	Prospective cohort study (single center)	N = 269<br>-daily fasters: n = 31<br>-non-HD days fasters: n = 102<br>- non-fasters: n = 136	45.7%	1.daily fasting<br>2. partial fasting: fasting on non-HD days<br>3. non-fasting<br><br>/16 hour	Not reported (no protocol specified)	Not reported (no protocol specified)	Not reported	-Significant increase in adjusted IDWG in daily and partial fasting group<br>-Significant rise in serum K in full fasting group<br>-Slight increase in serum creatinine in partial fasting group<br>-Stable albumin, calcium, & PO4
Megahed et al. (2019)/ Egypt	Multicenter cross-sectional observation (25 centers)	N = 2,055 (Fasters 46.96%)	18.9%	1.Frequent group (>17 days)<br>2.Infrequent group (≤17 days, non-HD days)<br>3. Non-fasting<br><br>/16 hour	Subjective self-reported symptom questionnaires	Not reported (no protocol specified)	Subjective hypo-glycemia significantly higher in fasters:<br>Group1: 10.2%<br>Group 2: 8.2%<br>Group 3:5.0%	-IDH higher in fasters<br>-Stable DW<br>-Significant increase in albumin, PO4, & Cr<br>- lower mortality in fasters driven by younger age and fewer comorbidities
Adanan et al. (2020)/ Malaysia	Prospective observational cohort study (3 centers)	N = 87 maintenance HD fasters	40.2%	Voluntary fasting (median fasting: 28 days)<br>/ 14 hour	Self-reported symptomatic recall	Not reported (no protocol specified)	Self-reported hypoglycemic symptoms: 15.7 %	-Reversible declines in BMI, IDWG, waist circumference, and fat mass with preserved lean mass<br>- Improved handgrip strength, -Reduced PO4, albumin, urea, and Cr<br>-Stable BP, lipids, and hsCRP
Emara et al. (2022) /Egypt	Prospective cohort study (single center)	N = 199<br>Complete: 14.1%,<br>Partial: 44.2%<br>Non-fasting: 41.7%	11.6%	Complete vs. Partial vs. Non-fasting<br>/15 hour	Not reported (no protocol specified)	Not reported (no protocol specified)	Not reported	-Low intradialytic complications in fasters<br>- Significant reduction in DW in complete and partial fasters
Qureshi et al. (2025) /Pakistan	Quasi-experimental study (single center)	N = 132<br>Complete: 5.3%,<br>Intermittent: 18.2%,<br>Non-fasting: 76.5%	Not reported	-Complete (daily)<br>-Intermittent (non-HD days)<br>- Non-fasting	Not reported (no protocol specified)	Not reported (no protocol specified)	Not reported	-Complete fasters: Stable UF, K, PO4, DW, and albumin<br>- Intermittent fasters: Significant increase in UF and K

Abbreviations, BP; blood pressure, Cr; creatinine, DM; diabetes mellitus, DW; dry weight, HbA1c; glycated hemoglobin, HD; hemodialysis, hs-CRP; high sensitivity C-reactive protein, IDH; intradialytic hypotension, IDWG; interdialytic weight gain, K; potassium, Kt/V; dialyzer clearance of urea normalized to total body water, PO4; phosphorus, PRN; pro re nata (as need), PTH; parathyroid hormone.
